# Study on the Hepatoprotective Effect Mechanism of Polysaccharides from Charred *Angelica sinensis* on the Layer Chickens Based on the Detection of the Intestinal Floras and Short-Chain Fatty Acids of Cecal Contents and Association Analysis

**DOI:** 10.3390/vetsci10030224

**Published:** 2023-03-15

**Authors:** Fanlin Wu, Peng Ji, Yonghao Hu, Chenchen Li, Jian He

**Affiliations:** College of Veterinary Medicine, Gansu Agricultural University, Lanzhou 730070, China; wufl@gsau.edu.cn (F.W.); huyongh@gsau.edu.cn (Y.H.); licc@st.gsau.edu.cn (C.L.); hej@st.gsau.edu.cn (J.H.)

**Keywords:** polysaccharides from charred *Angelica sinensis*, liver injury, layer chickens, intestinal floras, short-chain fatty acids

## Abstract

**Simple Summary:**

This study aimed to analyze the intervention mechanism of polysaccharides from charred *Angelica sinensis* (CASP) on the liver injury caused by Ceftiofur sodium and lipopolysaccharide from the perspective of the intestine. The detection of the intestinal floras and the short-chain fatty acids (SCFAs) of chicken cecal contents and their association analysis were carried out. The results showed that the structure of chicken liver in the normal control group was normal, while that in the model group was damaged. The structure of chicken liver in the CASP intervention group was similar to the normal control group. The intestinal floras in the model group were maladjusted compared to the normal control group. After the intervention of CASP, the diversity, and richness of chicken intestinal floras changed. The contents of acetic acid, butyric acid, and total SCFAs in the intervention group of CASP were significantly lower than those in the model group, and the contents of propionic acid and valeric acid in the intervention group of CASP were significantly lower than those in the model group and normal control group. The changes in the intestinal floras were correlated with the changes SCFAs in the cecum. It is confirmed that the liver-protecting effect of CASP is indeed related to the changes in intestinal floras and SCFAs content in the cecum.

**Abstract:**

To analyze the intervention mechanism of polysaccharides from charred *Angelica sinensis* (CASP) on the liver injury caused by Ceftiofur sodium (CS) and lipopolysaccharide (LPS) from the perspective of the intestine. Ninety-four one-day-old laying chickens underwent free feeding and drinking water for three days. Then, fourteen laying chickens were randomly selected as the control group, and sixteen laying chickens were selected as the model group. Sixteen laying chickens in the rest were randomly selected as the intervention group of CASP. Chickens in the intervention group were given CASP by the oral administration (0.25 g/kg/d) for 10 days, the control and model groups were given the same amount of physiological saline. During the 8th and 10th days, laying chickens in the model and CASP intervention group were subcutaneously injected with CS at the neck. In contrast, those in the control group were subcutaneously injected with the same amount of normal saline simultaneously. Except for the control group, the layer chickens in the model and CASP intervention groups were injected with LPS after CS injection on the 10th day of the experiment. In contrast, those in the control group were injected with the same amount of normal saline at the same time. 48 h after the experiment, the liver samples of each group were collected, and the liver injury was analyzed by hematoxylin-eosin (HE) staining and transmission electron microscopy. And the cecum contents of six-layer chickens in each group were collected, and the intervention mechanism of CASP on the liver injury from the perspective of the intestine was analyzed by the 16S rDNA amplicon sequencing technology and the short-chain fatty acids (SCFAs) detection of cecal contents based on Gas Chromatography-Mass Spectrometry (GC-MS), and their association analysis was carried out. The results showed that the structure of chicken liver in the normal control group was normal, while that in the model group was damaged. The structure of chicken liver in the CASP intervention group was similar to the normal control group. The intestinal floras in the model group were maladjusted compared to the normal control group. After the intervention of CASP, the diversity, and richness of chicken intestinal floras changed significantly. It was speculated that the intervention mechanism of CASP on the chicken liver injury might be related to the abundance and proportion of Bacteroidetes and Firmicutes. Compared with the model group, the indexes of ace, chao1, observed species, and PD whole tree of chicken cecum floras in the intervention group of CASP were significantly increased (*p* < 0.05). The contents of acetic acid, butyric acid, and total SCFAs in the intervention group of CASP were significantly lower than those in the model group (*p* < 0.05), and the contents of propionic acid and valeric acid in the intervention group of CASP were significantly lower than those in the model group (*p* < 0.05) and normal control group (*p* < 0.05). The correlation analysis showed that the changes in the intestinal floras were correlated with the changes in SCFAs in the cecum. It is confirmed that the liver-protecting effect of CASP is indeed related to the changes in the intestinal floras and SCFAs content in the cecum, which provides a basis for screening liver-protecting alternative antibiotics products for poultry.

## 1. Introduction

Chicken Colibacillosis (CC) is a common and important infectious disease that seriously endangers the health of chicks. Previous studies have found that the coexistence of Ceftiofur sodium (CS) and lipopolysaccharide (LPS) can cause liver damage in laying hens after curing CC with CS [1].

It is confirmed that the liver protective effect of polysaccharides from charred *Angelica sinensis* (CASP) is accurate, and its intervention mechanism is related to arachidonic acid metabolic and mTOR pathway [2], but it is not clear whether it is related to the changes of intestinal floras and the short-chain fatty acids (SCFAs) content in the cecum.

The intestinal floras are interdependent and restricted, forming a relatively stable microecology. There is a certain correlation between intestinal floras disorder and the mechanism of liver injury [3]. Some studies have confirmed that intestinal floras could affect the physiological and pathological processes of the liver in many ways [4,5,6]. The interaction between intestinal floras and the host immune system or other types of cells could induce liver inflammation, steatosis, and fibrosis. It is very important to deeply study the mechanism of specific intestinal microorganisms in the process of liver injury and lesion repair [7].

SCFAs are closely related to intestinal floras and can affect intestinal function [8,9]. With the change of floras structure, the species and quantity of short-chain fatty acids will also change. Some scholars have tried to explore the pathogenesis and drug intervention mechanism of liver injury by studying the changes in intestinal floras and short-chain fatty acid content. Wu [10] found mussel polysaccharide “α-D-glucan” could reshape the ecological distribution of intestinal floras in high-fat diet model rats, improve the abundance of some probiotics of intestinal floras, reduce the abundance of some intestinal pathogenic microorganisms, and exert the liver protection effect by regulating SCFAs. Jin [11] found that studying the changes in cecum floras and SCFAs content helped explain the intervention mechanism of probiotics on LPS-induced liver injury. Yu et al. [12] found that Chinese herbal compound microecological agents could maintain intestinal health and protect the liver by promoting the reproduction of *Lactobacillus* in the cecum of broilers. Zhang [13] found that okra powder could alleviate obesity symptoms and liver injury by regulating abnormal blood glucose and the disorder of lipid metabolism in high-fat model mice, and effectively regulating the imbalance of intestinal floras. And the results showed okra seed oil could effectively antagonize the increase of Aspartate Transaminase, Alanine aminotransferase, and Malondialdehyde and increase the activities of Glutathione peroxidase and Total superoxide dismutase in the liver, improve the liver injury induced by alcohol in mice, and effectively intervene in the imbalance of main intestinal microorganisms. Zhu et al. [14] found that the liver protection mechanism of Xiaozhi decoction might be related to the increase of *Lactobacillus* and *Bifidobacterium* in the intestine. In addition, Xiaozhi decoction could improve the inflammatory reaction of hepatocytes induced by free fatty acids. Therefore, the study on the changes of floras and SCFAs content in the chicken cecum helps explore the liver protective mechanism of CASP.

In this study, the liver histological observation was performed, 16S rDNA amplicon sequencing technology was used to screen the differential floras in cecal contents, GC-MS was used to detect the content of SCFAs, and the liver protection mechanism of CASP was analyzed from the perspective of the intestine through correlation analysis.

## 2. Materials and Methods

### 2.1. The Animal Experiment Program, Sampling, and Liver Histological Observation

The experimental protocol was approved by the Laboratory Animal Management Committee of Gansu Agricultural University. Ninety-four laying chickens (one-day-old) (Beijing Huadu Yukou Poultry Industry Co., Ltd., Beijing, China) underwent adaptive feeding 3d. Fourteen chickens were randomly selected as the control group, and sixteen from the model group. Sixteen were randomly selected as the intervention group of CASP (self-made, 0.25 g/kg/d). The above doses were based on the pre-experiment results [15]. Chickens in the intervention group were given CASP by the oral administration (0.25 g/kg/d) for 10 days, the next two groups were given the same amount of physiological saline. Chickens in the model and intervention group were subcutaneously injected with Ceftiofur sodium (Qilu Animal Health Products Co., Ltd., Jinan, China, 5 mg/kg) at the neck on the 8th and 10th days. Chickens in the control group were injected with the same amount of normal saline. Except for the control group, the chickens in the other groups were injected with self-made LPS (*Escherichia coli* O78, 4 mL/kg) after CS injection on the 10th day of the experiment. Chickens in the control group were injected with the same amount of normal saline. 48 h after the experiment, the liver samples of each group were collected, and the liver injury was analyzed by HE staining (Olympus, Tokyo, Japan, DP71) and transmission electron microscopy (JEOL, Tokyo, Japan, JEM1400FLASH). And the cecum contents of six chickens in each group were collected and frozen at −80 °C.

### 2.2. Sequencing and Analysis of 16S rDNA Amplicon of Cecum Content Floras

#### 2.2.1. DNA Extraction

The samples were extracted by the SDS method, and then the purity and concentration of DNA were detected by agarose gel electrophoresis. Finally, the DNA was diluted to 1 ng/μL with aseptic water standby.

#### 2.2.2. PCR Amplification

Primers were synthesized using diluted DNA as a template (Sangon biotech, Shanghai, China). PCR reaction system and procedure (30 μL): Phusion Master Mix (2×) 15 μL (New England Biolabs, East Hanover, NJ, USA), Primer (New England Biolabs, 2 μM) 3 μL, gDNA (New England Biolabs, 1 ng/μL) 10 μL, H_2_O (2 μL); Reaction procedure: pre denaturation at 98 °C for 1 min; 30 cycles including (98 °C, 10 s; 50 °C, 30 s; 72 °C, 30 s); 72 °C, 5 min. PCR products were made detection by electrophoresis with agarose gel (Sigma-Aldrich, St. Louis, MO, USA, concentration of 2%). Then it was detected with a Gradient PCR instrument (Bio-rad, Berkeley, CA, USA, T100).

#### 2.2.3. Mixing and Purification of PCR Products

With the Gel DNA recovery kit (Thermo Scientific, Waltham, MA, USA), the equal concentration mixing was carried out according to the final concentration of PCR products, and a 1× TAE (Solarbio, Beijing, China) concentration of 2% agarose gel electrophoresis was used for purification. Finally, the target band was recovered.

#### 2.2.4. Library Construction and Computer Sequencing

The library construction was completed according to the DNA library construction kit (Illumina, San Diego, CA, USA), and then Illumina Novaseq 6000 detection platform was used to complete the sequencing after Qubit quantification and library detection.

#### 2.2.5. Information Analysis Process

Firstly, data splicing and quality control analysis was carried out with FLASH software; Uparse was used to cluster the samples with Operational taxonomic units (OTUs), and Greengenes was used for species annotation; Then, abundance, Alpha diversity, Venn diagram, and Principal co-ordinates analysis (PCoA) were carried out for OTUs. Anosim and STAMP were used to analyze the differences in microbial community structure among groups to analyze species composition and diversity.

### 2.3. Short-Chain Fatty Acid Targeted Metabolomics Research Methods

#### 2.3.1. Standards Preparation

Accurately weighed the standard of acetic acid (National Medicine ≥ 99.5%), propionic acid (TCI > 99.0%), butyric acid (TCI > 99.0%), isobutyric acid (National Medicine > 99.0%), valeric acid (TCI > 98.0%), isovaleric acid (TCI > 99.0%), and caproic acid (Aladdin ≥ 99.5%), and used ethyl acetate (Merck, Kenilworth, NJ, USA, GC-MS grade) to prepare the mixed standard solution with the concentration of 0.1, 0.5, 1, 5, 10, 20, 50 and 100 μg/mL. Take 600 μL standard solution, add 25 μL of 4-methyl valeric acid as internal standard (final concentration was 500 μM), carried out Gas Chromatography-Mass Spectrometry analyzer (GC-MS, Agilent, Santa Clara, CA, USA, 7890A/5975C) detection, and the injection volume was 1 μL, shunt ratio 10:1, shunt injection.

#### 2.3.2. Metabolite Extraction

30 mg of samples were taken with Electronic balance (WT3003N), and 0.5% phosphoric acid (National Medicine, Beijing, China, 900 μL) was added and resuspended, shaken, and mixed evenly for 2 min with the Vortex instrument (QL-866), centrifuged 14,000× *g* for 10 min with the Refrigerated centrifuge (Eppendorf, Hamburg, Germany, 5430R). Then the supernatant of 800 μL was taken, the same amount of ethyl acetate (Merck, GC-MS grade) was added, shaken, and mixed for 2 min with the Vortex instrument (QL-866), and centrifuged 14,000× *g* for 10 min with the Refrigerated centrifuge (Eppendorf, 5430R); The upper organic phase of 600 μL was taken, and 4-methyl valeric acid was added as the internal standard (the final concentration was 500 μM), at the end 1 μL was taken after mixing and was detected by GC-MS with the shunt ratio of 10:1.

#### 2.3.3. QC Samples Preparation

QC samples were prepared with all the samples mixed equally, which were used to investigate the stability of the detection process.

#### 2.3.4. GC-MS Analysis

GC conditions: DB-WAX capillary column (Agilent, 30 m × 0.25 mm ID × 0.25 μm). Programmed temperature rise: initially 90 °C, raise the temperature to 120 °C at 10 °C/min, then rise to 150 °C (5 °C/min), finally increase the temperature to 250 °C at 25 °C/min, and maintain for 2 min. The carrier gas was helium, with a carrier gas flow rate of 1.0 mL/min.

MS conditions: the temperature of the injection port was 250 °C; the Ion source temperature was 230 °C; The temperatures of the quadrupole and transmission line were 150 °C and 250 °C respectively, and the electronic energy was 70 eV.

#### 2.3.5. Data Processing

The retention time and peak area of the chromatogram of different standards were extracted. The standard curve was established according to the concentration and peak area, and then the SCFAs contents in different groups of the cecal content samples were calculated.

### 2.4. Combined Analysis of Intestinal Floras and Short-Chain Fatty Acids

For the differential floras and short-chain fatty acids screened in the early stage, the spearman method was used for correlation analysis. R language and Cytoscape software were used to carry out matrix heatmap, hierarchical clustering, and correlation network analysis. The interaction relationship between cecal microbial floras and metabolites was explored from multiple angles.

## 3. Results

### 3.1. Liver Histological Observation

#### 3.1.1. HE Staining

**Figure 1:**
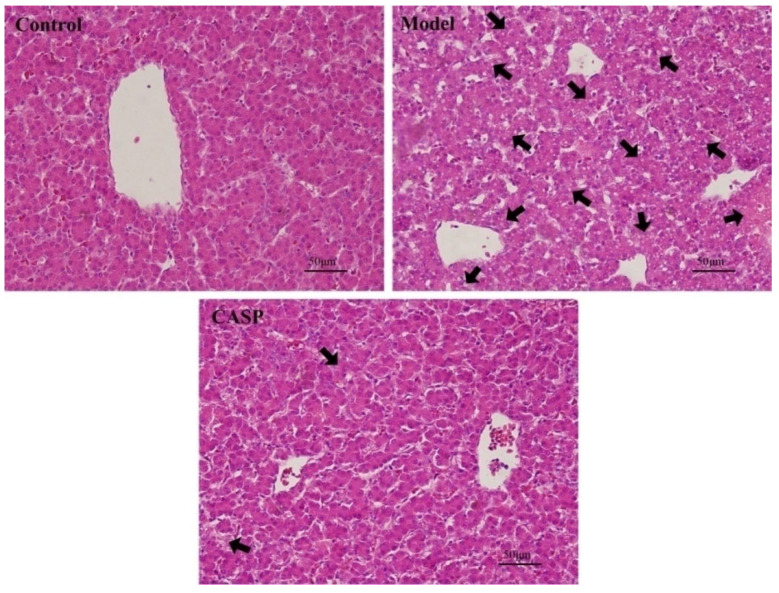
Pathological observation results of chicken liver of each group (HE, 400×). Note: The black arrows refer to the obvious hepatic lesions.

The histopathological observation results of HE staining of chicken liver in each group are shown in Figure 1.

It could be seen that compared with the normal control group, the chicken liver in the model group had severe liver cell necrosis, difficult identification of liver lobules, structural disorder, nuclear pyknosis, extensive vacuolar degeneration, fatty degeneration, and incomplete cell structure. Compared with the model group, the chicken liver cell structure of the CASP intervention group was more complete, the degeneration and necrosis of liver cells were reduced, and the arrangement was more regular. It can be seen that CASP has a definite effect on the chicken liver damage caused by CS and LPS.

#### 3.1.2. Transmission Electron Microscopy

**Figure 2:**
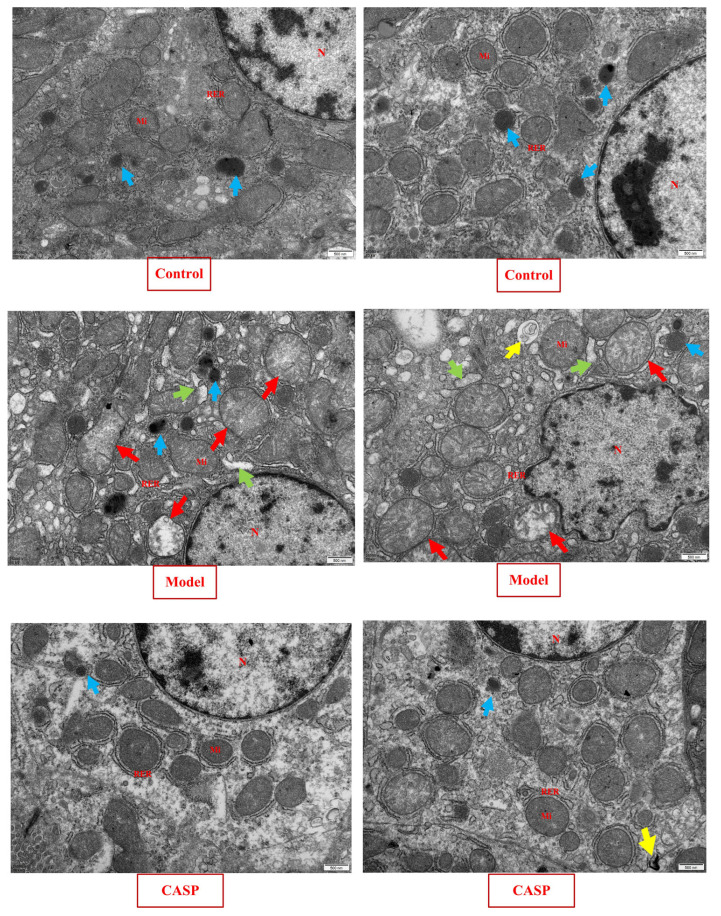
Transmission electron microscopy observation results of chicken liver in each group (25,000×). Note: Nucleus (N), Mitochondria (Mi), Rough endoplasmic reticulum (RER), Primary lysosome (↑), Rough endoplasmic reticulum dilates (↑), Mitochondrial swelling (↑), autophagy (↑).

The transmission electron microscopic observation results of chicken livers in each group are shown in Figure 2.

In the normal control group, the morphology and structure of the hepatocytes in the chicken liver were normal, the structure of the organelles in the cytoplasm was complete and clear, no obvious lesions were found, and a small amount of primary lysosomes were occasionally seen. In the model group, the liver nuclei in the chicken liver were mostly irregular, with chromatin aggregation. Most of the rough endoplasmic reticulum in the cytoplasm had expanded, a large number of mitochondria have obvious swelling (crista dissolution and fracture), and a large number of primary lysosomes and obvious autophagy could also be seen. It could be found that after modeling with CS and LPS, chicken hepatocytes had significant pathological changes, the endoplasmic reticulum and mitochondria have been damaged, and autophagic bodies have appeared, which confirms that autophagy is indeed involved in the pathological process of liver injury caused by CS and LPS. The chicken liver morphology and structure of liver cells in the intervention group of CASP were similar to that in the normal control group, only a small amount of autophagy was found in the cytoplasm. It could be found that CASP had a good intervention effect on the pathological process of chicken liver injury caused by CS and LPS.

### 3.2. Analysis Results of Intestinal Floras

#### 3.2.1. 16S rDNA Amplicon Sequencing Data Preprocessing and Quality Control Statistics

The quality control results of the samples are listed in Table 1, which showed that this method was stable and feasible.

#### 3.2.2. Analysis Results of Richness and Uniformity of Samples in Each Group

**Figure 3:**
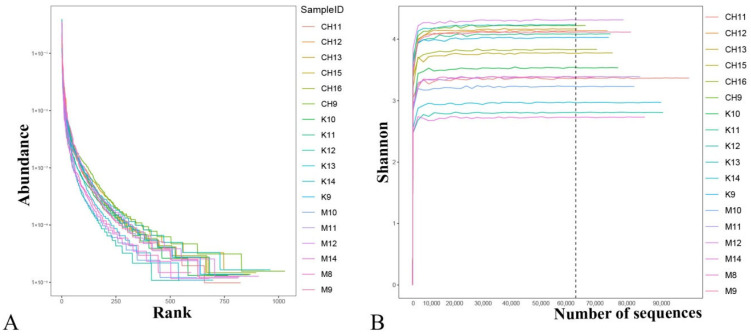
Analysis of uniformity and richness of species in samples. Note: (**A**) Rank abundance curve; (**B**) Shannon curves; K refers to the normal control group, CH refers to the CASP intervention group, and M refers to the model group. The same as below.

The sample species have good uniformity and richness (Figure 3A). The sequencing data was reasonable, and more data would not produce new species (Figure 3B).

#### 3.2.3. Venn Analysis of OTUs Distribution in Cecum Content Floras

**Figure 4:**
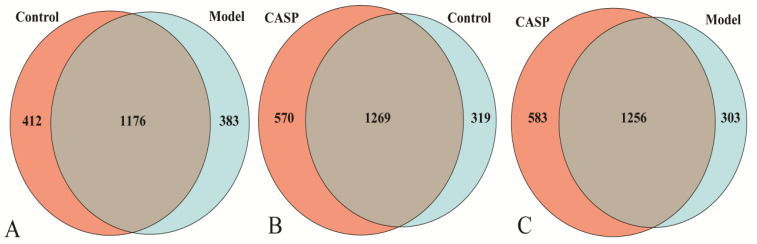
Venn diagram of OTUs distribution in each group. Note: (**A**) OTUs Venn diagram of the normal control group and the model group; (**B**) OTUs Venn diagram of the normal control group and the intervention group of CASP; (**C**) OTUs Venn diagram of the model group and the intervention group of CASP.

The results of OTUs clustering of cecal microorganisms with 97% consistency are shown in Figure 4. The middle gray area was the common OTUs between the two groups.

#### 3.2.4. Relative Abundance Analysis Results of Cecum Content Floras

##### Relative Abundance of Floras Distribution at the Phylum Level

**Figure 5:**
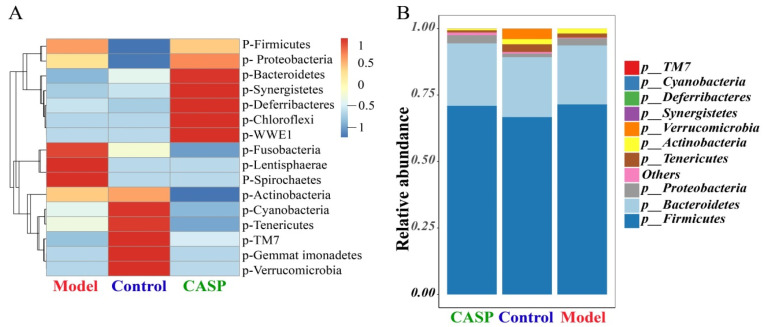
Relative abundance of floras at phylum level of chicken cecal contents in each group. Note: (**A**) Cluster analysis diagram of relative abundance; (**B**) Relative abundance diagram of floras distribution.

The distribution of intestinal floras at the phylum level of each group is shown in Figure 5. At the phylum level, the richness of Bacteroidetes, Synergistetes, Deferribacteres, Chloroflexi, and WWE1 in the intervention group of CASP was higher, and the richness of Actinobacteria was significantly lower (*p* < 0.05). In the model group, Fusobacteria, Lentisphaerae, and Spirochaetes had higher richness. In the normal control group, Cyanobacteria, Tenericutes, Gemmatimonadetes, TM7, and Verrucomicrobia had higher richness (Figure 5A). Firmicutes, Bacteroidetes, and Proteobacteria were the dominant floras in the cecum contents (Figure 5B).

##### Relative Abundance of Floras Distribution at the Genus Level

**Figure 6:**
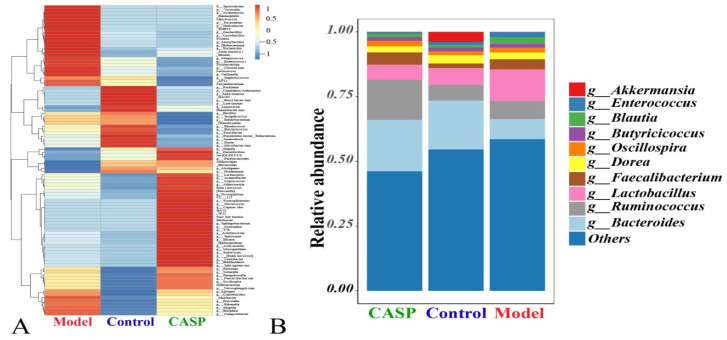
Relative abundance of chicken cecal contents in each group at the genus level. Note: (**A**) Cluster analysis diagram of relative abundance; (**B**) Relative abundance diagram of floras distribution.

The distribution of intestinal floras of each group at the genus level is shown in Figure 6. At the genus level, the richness of *Lachnospira*, *Acinetobacter*, *Coprococcus*, *Adlercreutzia*, *Ruminococcus*, *Prevotella*, *Novispirillu*, *Syntrophomonas*, *Micrococcus*, *Cupriavidu*, *HA73*, *W22*, *Succinatimonas*, *Pelobacter*, *Sphingobacterium*, *Syntrophus*, *T78*, *Allobaculum*, *Spirosoma*, *Herbaspirillum*, *Asticcacaulis*, *Mucispirillum*, *Rubrivivax*, *Caulobacter*, *Burkholderia*, *Sphingomonas*, *Ralstonia*, *Sutterella*, *Paraprevotella*, *Faecalibacterium*, *Oscillospira*, *Gallibacterium*, and *Novosphingobium* in the intervention group of CASP was higher. In the model group, the richness of *Sporosarcina*, *Victivallis*, *Actinomyces*, *Haemophilus*, *Macrococcus*, *Treponema*, *Helicobacter*, *SMB53*, *Geobacillus*, *Lactobacillus*, *Proteus*, *Anoxybacillus*, *Bubacterium*, *Escherichia*, *Anaerotruncus*, *Blautia*, *Streptococcus*, *Enterococcus*, *Fusobacterium*, *Clostridium*, *Lactococcus*, *Veillonella*, *Staphylococcus*, *AF12*, and *Corynebacterium* were higher. In the normal control group, the richness of *Facklamia*, *Candidatus Arthromitus*, *Akkermansia*, *DA101*, *Brevibacterium*, *Luteimonas*, *Dehalobacterium*, *Bacillus*, *Jeotgalicoccus*, *Bifidobacterium*, *Granulicatella*, *Rhodococcus*, *Butyricicoccus*, *Turicibacter*, *Pseudoramibacter*, *Eubacterium*, *Anaerofustis*, and *Microbacterium* were higher (Figure 6A). At the genus level, *Bacteroides*, *Ruminococcus*, and *Lactobacillus* were the top three dominant floras (Figure 6B).

#### 3.2.5. The α Diversity Analysis of Cecum Content Floras in Each Group

**Figure 7:**
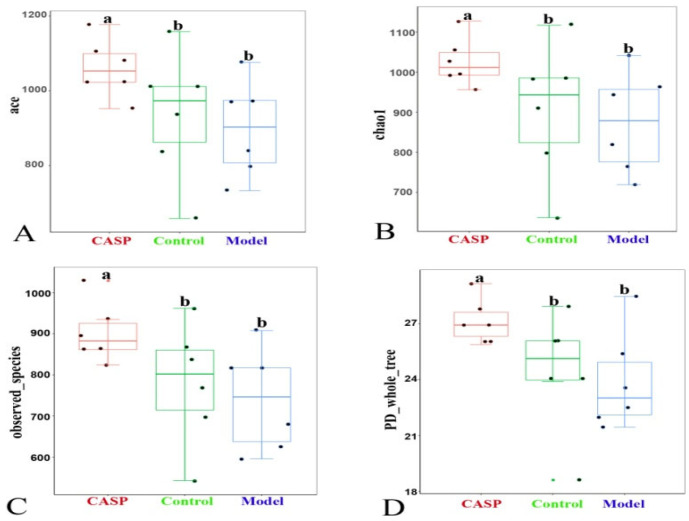
The α-diversity analysis of cecum content floras in each group. Note: (**A**) Box chart of ace index difference between groups; (**B**) Box chart of Chao 1 index difference between groups; (**C**) Box chart of observed species index difference between groups; (**D**) Box chart of PD whole tree index difference between groups. The same letter, and the different letter indicate that the inter-group differences difference is non-significant or significant, respectively.

The α-diversity analysis results of cecum content floras in each group (Figure 7) showed that compared with the normal control group, the ace, chao1, observed species, and PD whole tree indexes of cecum floras in liver injury chicken decreased, and the differences were not significant, prompting that the biodiversity of intestinal floras would be reduced when the antibiotics were used. Compared with the model group, the ace, chao1, observed species, and PD whole tree indexes of chicken cecum floras in the intervention group of CASP were significantly increased (*p* < 0.05).

#### 3.2.6. PCoA Analysis of Cecum Content Floras in Each Group

**Figure 8:**
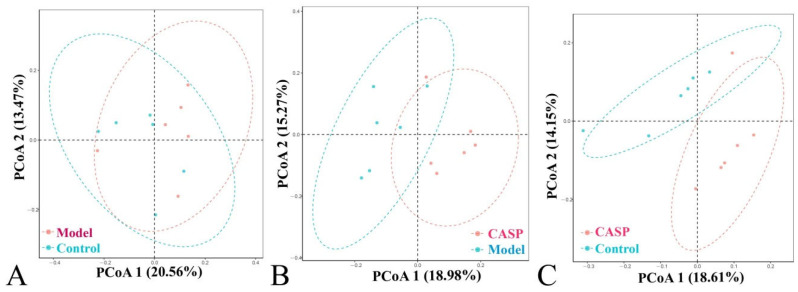
Principal coordinate analysis of cecum content floras in each group. Note: (**A**) PCoA diagram of the normal control group and the model group; (**B**) PCoA diagram of the model group and the intervention group of CASP; (**C**) PCoA diagram of the normal control group and the intervention group of CASP.

The community structure differences of different groups were shown in the PCoA analysis chart (Figure 8).

#### 3.2.7. Analysis of Differential Floras of Cecal Contents in Each Group

##### Anosim Analysis of Cecum Content Floras in Each Group

**Figure 9:**
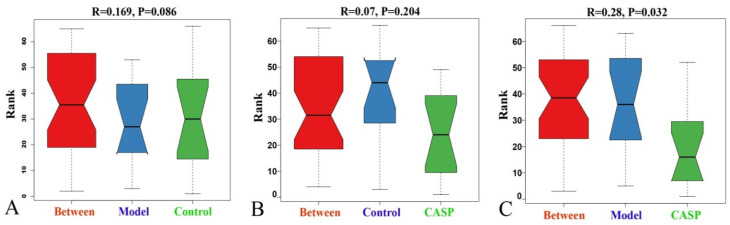
Anosim analysis of cecum content floras in each group. Note: (**A**) Anosim analysis diagram of the normal control group and the model group; (**B**) Anosim analysis diagram of the normal control group and the intervention group of CASP; (**C**) Anosim analysis diagram of the model group and the intervention group of CASP.

Anosim analysis is a nonparametric test method based on the Bray-Curtis algorithm, which is mainly used to test whether the difference between groups is significantly greater than that within groups. As shown in Figure 9, there was a significant difference between the intervention group of CASP and the model group (r = 0.28, *p* < 0.05), indicating a significant difference in the structure of chicken cecum floras between the two groups.

##### STAMP Difference Analysis of Cecum Content Floras in Each Group

**Figure 10:**
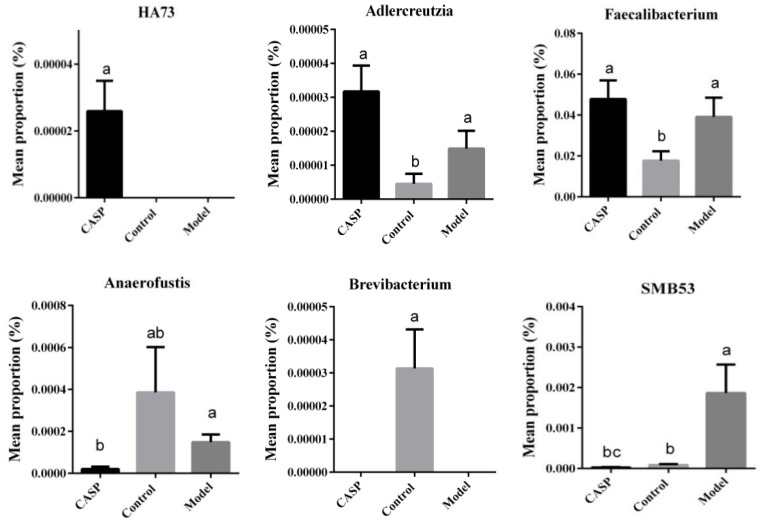
STAMP difference analysis of cecum content floras in each group. Note: The same letter, and the different letter indicate that the inter-group differences difference is non-significant or significant, respectively.

The results of the STAMP difference analysis at the genus level are shown in Figure 10. 

At the genus level, the abundances of *Adlercreutzia* and *Faecalibacterium* in the intervention group of CASP were significantly higher than those in the normal control group, and the abundance of *HA73* was significantly higher than that in the normal control group and the model group (*p* < 0.05). The abundance of *SMB53* in the intervention group of CASP was significantly lower than that in the normal control group and model group (*p* < 0.05), the abundance of *Brevibacterium* was significantly lower than that of the normal control group (*p* < 0.05), and the abundance of *Anaerofustis* was significantly lower than that of the model group (*p* < 0.05).

#### 3.2.8. KEGG Function Prediction

**Figure 11:**
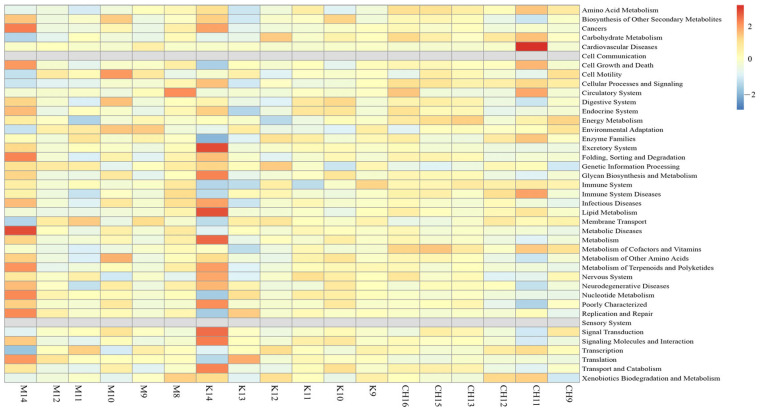
Prediction results of KEGG function of cecum content floras in each group. Note: K refers to the normal control group, CH refers to the CASP intervention group, and M refers to the model group. The same as below.

The KEGG function prediction results of cecum content floras in each group are shown in Figure 11. 

In Figure 11, it could be seen that the model group was mainly enriched in metabolic diseases, cell growth, and death, and the intervention group of CASP was mainly enriched in amino acid metabolism, cell growth, and death, and the immune system.

### 3.3. Analysis Results of Short-Chain Fatty Acids

#### 3.3.1. The Detection of SCFAs in Cecal Contents of Chickens in Each Group

**Figure 12:**
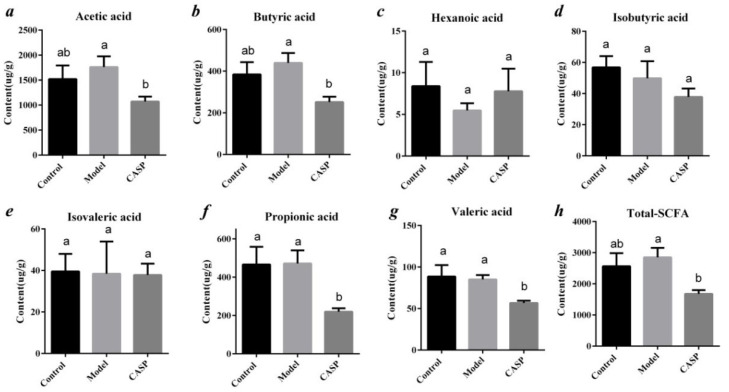
Content of SCFAs in chicken cecum contents in each group. Note: (***a***) The contents of acetic acid of different groups; (***b***) The contents of butyric acid of different groups; (***c***) The contents of hexanoic acid of different groups; (***d***) The contents of isobutyric acid of different groups; (***e***) The contents of isovaleric acid of different groups; (***f***) The contents of propionic acid of different groups; (***g***) The contents of valeric acid of different groups; (***h***) The contents of total-SCFA of different groups. The same letter, and the different letter indicate that the inter-group differences difference is non-significant or significant, respectively.

As shown in Figure 12, the contents of acetic acid, butyric acid, and total SCFAs in the intervention group of CASP were significantly lower than those in the model group (*p* < 0.05), and the contents of propionic acid and valeric acid were significantly lower than those in model group (*p* < 0.05) and normal control group (*p* < 0.05).

#### 3.3.2. Heatmap Analysis between SCFAs and Different Intestinal Floras of Chicken Cecum Contents

**Figure 13:**
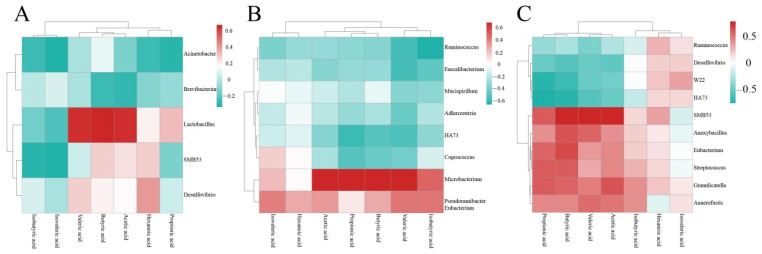
Heatmap of SCFAs and intestinal floras of chicken cecum contents of different groups. Note: (**A**) Cluster heatmap of SCFAs and intestinal floras in the normal control group and the model group; (**B**) Cluster heatmap of SCFAs and intestinal floras in the normal control group and the intervention group of CASP; (**C**) Cluster heatmap of SCFAs and intestinal floras in the model group and the intervention group of CASP.

The functional abundance value can be directly expressed with colors change in the heatmap. Heatmap analysis was performed on the results of SCFAs and differential intestinal floras in cecum contents. The results are shown in Figure 13. 

*Lactobacillus* was closely related to butyric acid, acetic acid, and valeric acid in the normal control and model groups. In the normal control group and the intervention group of CASP, the relationship between *Microbacterium* and acetic acid, propionic acid, butyric acid, and valeric acid was close. *SMB53*, *Anoxybacillus*, *Eubacterium*, *Streptococcus*, *Granulicatella*, and *Anaerofustis* in the model group and the intervention group of CASP were closely related to propionic acid, valeric acid, butyric acid, and acetic acid, and isobutyric acid.

### 3.4. Correlation Analysis of Intestinal Floras and SCFAs in Chicken Cecum Contents

#### 3.4.1. Correlation Analysis of Intestinal Floras and SCFAs in Chicken Cecum Contents between the Normal Control Group and the Model Group

**Figure 14:**
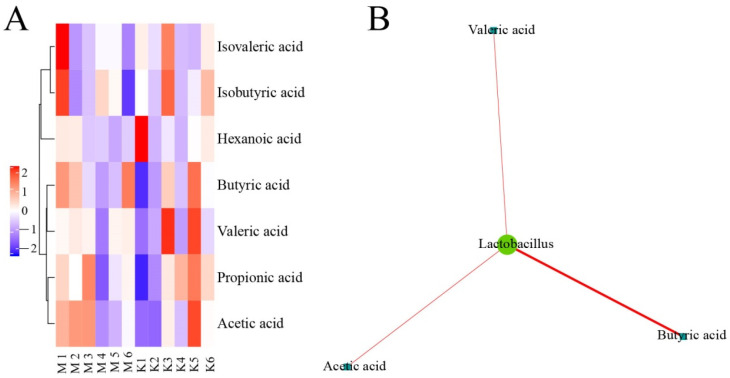
Correlation analysis of intestinal floras and SCFAs in chicken cecum contents between the normal control group and the model group. Note: (**A**) Cluster heatmap of SCFAs between the normal control group and the model group; (**B**) Network of intestinal floras and SCFAs between the normal control group and the model group.

As shown in Figure 14, *Lactobacillus* was positively correlated with the production of valeric acid, acetic acid, and butyric acid in the normal control group and the model group.

#### 3.4.2. Correlation Analysis of Intestinal Floras and SCFAs in Chicken Cecum Contents between the Normal Control Group and the Intervention Group of CASP

**Figure 15:**
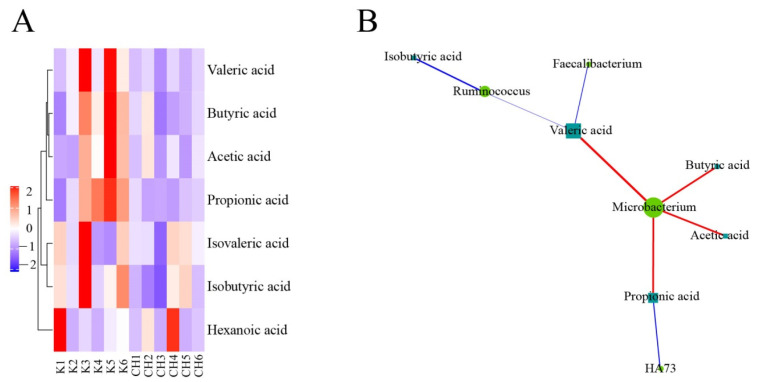
Correlation analysis of intestinal floras and SCFAs in chicken cecum contents between the normal control group and the intervention group of CASP. Note: (**A**) Cluster heatmap of SCFAs between the normal control group and the intervention group of CASP; (**B**) Network of intestinal floras and SCFAs between the normal control group and the intervention group of CASP. The red and blue line represents positive and negative correlation, respectively. The same as below.

As shown in Figure 15, *Microbacterium* in the normal control group and the intervention group of CASP was positively correlated with the production of butyric acid, acetic acid, propionic acid, and valeric acid. *HA73* was negatively correlated with the production of propionic acid. *Ruminococcus* was negatively correlated with the production of isobutyric acid and valeric acid. *Faecalibacterium* was negatively associated with the production of valeric acid.

#### 3.4.3. Correlation Analysis of Intestinal Floras and SCFAs in Chicken Cecum Contents between the Model Group and the Intervention Group of CASP

**Figure 16:**
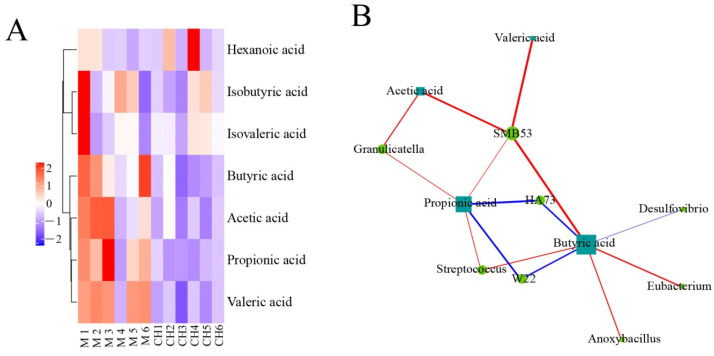
Correlation analysis of intestinal floras and SCFAs in chicken cecum contents between the model group and the intervention group of CASP. Note: (**A**) Cluster heatmap of SCFAs between the model group and the intervention group of CASP; (**B**) Network of intestinal floras and SCFAs between the model group and the intervention group of CASP.

As shown in Figure 16, in the model group and the intervention group of CASP, *SMB53* in the model group and the intervention group of CASP was positively correlated with the production of valeric acid, acetic acid, butyric acid, and propionic acid; *Granulicatella* was positively correlated with the production of acetic acid and propionic acid; *HA73* was negatively correlated with the production of propionic acid and butyric acid; *Streptococcus* was positively related to the production of propionic acid and butyric acid; *W22* was negatively correlated with the production of propionic acid and butyric acid. In addition, butyric acid production was closely related to *Desulfovibrio*, *Eubacterium*, and *Anoxybacillus*.

## 4. Discussion

This study aimed to analyze the intervention mechanism of CASP on the liver injury caused by CS and LPS from the perspective of the intestine, the detection of the intestinal floras of chicken cecal contents by the 16S rDNA amplicon sequencing technology and the SCFAs detection of cecal contents based on GC-MS and their association analysis was carried out. Firstly, the results of liver histological observation with HE staining and transmission electron microscopy showed the liver injury modeling caused by CS and LPS was successful. HE staining and transmission electron microscopy are two kinds of direct and classic methods for judging liver injury. Qiu et al. [16] found that HE staining and transmission electron microscopy could be used to observe the occurrence and improvement of liver fibrosis. Feng et al. [17] found that HE staining and transmission electron microscopy could be used to observe the occurrence and improvement of liver fibrosis. And the results of liver histological observation also showed the polysaccharides could make resistance to liver injury induced by LPS. Xu et al. [18] found that *Dicliptera chinensis* (L.) Juss (Acanthaceae) polysaccharide could resist the liver injury induced by LPS. Li et al. [19] found that the Plantago seed polysaccharide could resist the liver injury induced by LPS.

In order to analyze the intervention mechanism of CASP on the liver injury from the perspective of the intestine, the 16S rDNA amplicon sequencing technology was carried out. Intestinal flora is closely related to liver injury [20].

Relative abundance analysis results of cecum content floras showed that at the phylum level, the richness of Bacteroidetes, Synergistetes, Deferribacteres, Chloroflexi, and WWE1 in the intervention group of CASP was higher, and the richness of Actinobacteria was significantly lower (*p* < 0.05), which was consistent with the previous research report [21]. Firmicutes were the largest group of bacteria in the intestinal floras, most of which were Gram-positive. Many of its members were beneficial bacteria. For example, *Lactobacillus* is probiotic, and the produced substances could improve the immunity of chickens [22]. In addition, Firmicutes also included such as *Streptococcus* and other pathogenic bacteria. In this study, the abundance of Firmicutes in chicken cecum in each group changed significantly. The increase of Bacteroidetes was positively correlated with the immunity of chickens [23]. In this study, the abundance of Bacteroidetes in chicken cecum contents decreased after liver injury. The abundance of Bacteroidetes in chickens increased significantly after being intervened by CASP, suggesting that CASP could improve the immunity of chickens. This study also showed that the abundance of TM7 decreased significantly after liver injury, suggesting that the digestive function of chickens with liver injury was affected. The abundance of TM7 in the intervention group of CASP was corrected. Liu [24] found that Fusobacteria in chicken intestinal were harmful and could inhibit the growth performance of chickens. Xing [25] found that the increase in Spirochaetes would affect chicken intestinal health and reduce intestinal digestion and absorption and mucosal immune function. Li [26] found that LPS stress could reduce the abundance of Tenericutes. Some studies have reported that the changes in the proportion of Bacteroidetes and Firmicutes would cause diseases [27,28,29]. This study found that the proportion of Bacteroidetes and Firmicutes in each group changed. Through comprehensive analysis, it was found that the intervention mechanism of CASP on chicken liver injury induced by CS and LPS may be related to the abundance of some harmful floras and beneficial floras and the proportion of Bacteroidetes and Firmicutes.

According to the relative abundance analysis, results of floras distribution at the genus level, *Bacteroides*, *Ruminococcus*, and *Lactobacillus* were the top three dominant floras. *Bacteroides*’ anaerobic respiration mainly produced acetic acid, isovaleric acid, and succinic acid, which could stimulate the inner wall of the intestine to produce fucosylated glycans. Fucosylation could regulate leukocyte adhesion, regulate the development and differentiation of immune cells such as macrophages, neutrophils, and B cells, and play an essential role in many inflammatory diseases [30]. In addition, *Bacteroides* could stimulate epithelial angiogenesis, thus enhancing nutrient absorption. However, the nonalcoholic fatty liver could reduce the proportion of *Bacteroides* in the microbiota composition, indicating that liver injury would affect the abundance of *Bacteroides* [31]. In this study, the abundance of *Bacteroides* in the chicken cecum of the model group was significantly lower than that of the normal control group and the CASP intervention group. *Ruminococcus*, one of the most effective bacteria for decomposing carbohydrates, played an essential role in stabilizing the intestinal barrier and increasing energy. *Ruminococcus* was also reported as a kind of beneficial bacterium [32]. *Oscillospira* was an anaerobic bacterium belonging to *Ruminococcus* in Firmicutes, which could ferment complex plant carbohydrates. This study found that the abundance of *Ruminococcus* and *Oscillospira* in the intervention group of CASP was higher. Hepaticus in *Helicobacter* could cause chronic liver injury in mice [33]. Another study also found that inflammatory bowel disease was closely related to *Helicobacter*, and showed that T cells played an essential role in this process [34]. The results of this study showed that the abundance of *Helicobacter* in the chicken cecum of the model group was higher. It was speculated that the liver injury caused by CS and LPS might be related to the high abundance of *Helicobacter* in the chicken’s cecum.

According to the α diversity analysis results of cecum content floras in each group, it may be confirmed that CASP could improve the microecological of chicken cecum content floras. And the PCoA analysis results of cecum content floras in each group also confirmed that there were differences in the coincidence degree of chicken cecum content floras in each group, indicating that after the liver injury induced by CS combined with LPS, the chicken cecum content floras changed, and the floras also changed after the intervention of CASP.

The STAMP difference analysis results of cecal content floras in each group confirmed that *Faecalibacterium* in the intervention group of CASP were significantly higher than those in the normal control group. According to the analysis result, *Faecalibacterium* may be related to the enhancement of liver metabolism and growth promotion [35]. The KEGG function prediction results showed that the liver injury was mainly enriched in metabolic diseases, cell growth, and death, and the intervention effect on the liver injury of CASP was mainly enriched in amino acid metabolism, cell growth, and death, and the immune system, which was consistent with the previous conclusions [2].

The results in Figure 12 also showed that total SCFAs in the intervention group of CASP were significantly lower than those in the model group (*p* < 0.05). The change trend of SCFAs in the cecal contents of chickens in each group was consistent with the change trend of SCFAs in the previous research [36]. It is in accordance with the results in Figure 5A. The results in Figure 5A showed that *Bacteroides* abundance in the chicken caecum contents of the intervention group of CASP was the highest and that in the model group was the lowest. *Bacteroides*, as a member of the polysaccharide degradation alliance, were the primary source of propionic acid. It was speculated that they might be consumed by degrading undigested polysaccharides in vivo. Therefore, the propionic acid in the chicken caecum contents of the intervention group of CASP was lower. It was speculated that SCFAs might maintain homeostasis and physiological metabolism through compensatory increase after liver injury. The liver injury of chicken in the intervention group of CASP was significantly repaired. It was speculated that many SCFAs were consumed to repair liver injury. Through enterohepatic circulation, SCFAs may be transferred from the intestine to the liver to play the effect of protecting liver. In addition, SCFAs were produced by the carbohydrates in the undigested and unabsorbed food residues through anaerobic fermentation in the intestine. After the liver injury, carbohydrates in the undigested and unabsorbed food residues in the intestine stagnated. In addition, the change in the floral structures led to the increase of SCFAs produced by fermentation. In the intervention group of CASP, the liver injury of chicken was significantly repaired, and the carbohydrates in the undigested and unabsorbed food residues in the intestine decreased, the content of SCFAs produced was reduced under the change of floras structure [37,38]. In addition, some studies also showed that the *Lactobacillus* mixture could regulate the intestinal floras, thereby increasing the production of SCFAs and reducing Gram-negative bacteria [39], which were consistent with the results in Figure 6.

The level of serum SCFAs increased in rats with nonalcoholic fatty liver disease, suggesting that SCFAs may be related to the pathogenesis of the nonalcoholic fatty liver disease [40]. Li et al. [41] also found the concentrations of isobutyric acid and isovaleric acid in the control group were higher than those before and after the treatment (*p* < 0.05). These results are consistent with those in this study. Another study found that the content of valeric acid and hexanoic acid in the stool of the non-alcoholic fatty liver fibrosis group was significantly higher than that of the healthy control group [42]. Another study found that the phytosterol ester intervention could reduce the level of short-chain fatty acids in the colon contents of rats with nonalcoholic fatty liver in varying degrees [43].

The correlation analysis results of intestinal floras and SCFAs showed *Lactobacillus* was positively correlated with the contents of valeric acid, acetic acid, and butyric acid in the normal control group and the model group, which was consistent with the previous literature reports [44]. Peng et al. [45] also found that the content of *Lactobacillus* in the chicken cecum was positively correlated with the content of acetic acid.

## 5. Conclusions

This study confirmed that CASP could protect the liver by improving the diversity of chicken intestinal floras, affect the abundance and proportion of Bacteroidetes and Firmicutes, and interfere with the content of SCFAs in chicken cecal by affecting the abundance of *Lactobacillus*, *SMB53*, and *Microbacillus*. It was speculated that CASP might intervene in the chickens’ liver injury induced by CS combined with LPS by restoring intestinal floras balance and affecting the content of SCFAs. This study provided the theoretical basis for screening the hepatoprotective drugs for chickens and ensuring the chicken’s health and food safety from the perspective of an intestinal angle. 

## Figures and Tables

**Table 1:** The quality control results of the samples.

Sample	Reads	Bases	Q20	Q20Rate	Q30	Q30Rate	GC	N
ZTPSN20BW097-K13	219,822	54,405,945	53,095,006	97.59	51,009,017	93.76	28,653,280	369
ZTPSN20BW098-K9	230,424	56,914,728	55,492,538	97.5	53,235,081	93.53	29,840,847	483
ZTPSN20BW099-K10	241,658	59,568,697	58,033,670	97.42	55,591,097	93.32	31,056,569	568
ZTPSN20BW100-K11	223,926	55,085,796	53,643,629	97.38	51,383,229	93.28	29,449,720	606
ZTPSN20BW101-K12	256,786	63,554,535	61,918,471	97.43	59,329,503	93.35	33,954,429	709
ZTPSN20BW102-K14	257,258	63,542,726	61,985,848	97.55	59,481,218	93.61	33,549,341	503
ZTPSN20BW103-M8	207,452	51,136,918	49,870,028	97.52	47,847,271	93.57	26,914,916	375
ZTPSN20BW104-M9	230,724	56,758,104	55,342,887	97.51	53,074,709	93.51	30,475,442	431
ZTPSN20BW105-M10	244,884	60,608,790	59,146,038	97.59	56,800,744	93.72	31,486,693	410
ZTPSN20BW106-M11	267,806	66,148,082	64,638,064	97.72	62,180,328	94	35,509,484	698
ZTPSN20BW107-M12	239,984	59,156,056	57,663,073	97.48	55,255,279	93.41	31,245,292	514
ZTPSN20BW108-M14	250,800	61,696,800	60,124,246	97.45	57,626,784	93.4	31,966,576	276
ZTPSN20BW109-CH9	232,454	57,532,365	56,130,840	97.56	53,912,004	93.71	30,513,417	569
ZTPSN20BW110-CH16	232,930	57,533,710	56,085,840	97.48	53,781,860	93.48	30,340,874	418
ZTPSN20BW111-CH11	248,776	61,323,284	59,739,475	97.42	57,195,183	93.27	32,461,557	524
ZTPSN20BW112-CH12	228,298	56,161,308	54,682,599	97.37	52,362,377	93.24	29,843,898	543
ZTPSN20BW113-CH13	210,354	52,062,615	50,702,981	97.39	48,587,756	93.33	27,086,986	571
ZTPSN20BW114-CH15	237,158	58,578,026	57,125,402	97.52	54,829,483	93.6	30,437,818	341

Note: K refers to the normal control group, CH refers to the CASP intervention group, and M refers to the model group. The same as below.

## Data Availability

The data and analyses presented in this paper are freely available with the corresponding author at a reasonable request.

## References

[1] Liu S.L., Ji P., Wei Y.M., He J., Li C.C., Hua Y.L., Yao W.L., Zhang X.S., Yuan Z.W. (2020). Establishment of liver injury model induced by ceftiofur sodium combined with Lipopolysaccharide. Prog. Vet. Sci..

[2] Wu F.L., Hu Y.H., Ji P., Li C.C., He J. (2022). Metabonomics study on the hepatoprotective effect mechanism of polysaccharides from different processed products of Angelica sinensis on layer chickens based on UPLC-Q/TOF-MS/MS, multivariate statistical analysis and conjoint analysis. Biomed. Chromatogr..

[3] Zhao J.H., Mao X.R. (2019). Mechanism of intestinal flora in the pathogenesis and treatment of nonalcoholic fatty liver disease. J. Lanzhou Univ. Med. Sci..

[4] Wang L., Cao Z.M., Zhang L.L., Li J.M., Lv W.L. (2022). The Role of Gut Microbiota in Some Liver Diseases: From an Immunological Perspective. Front. Immunol..

[5] Liu Q., Tian H., Kang Y., Tian Y., Li L., Kang X., Yang H., Wang Y., Tian J., Zhang F. (2021). Probiotics alleviate autoimmune hepatitis in mice through modulation of gut microbiota and intestinal permeability. J. Nutr. Biochem..

[6] Yin R., Liu S., Jiang X., Zhang X., Wei F., Hu J. (2022). The Qingchangligan Formula Alleviates Acute Liver Failure by Regulating Galactose Metabolism and Gut Microbiota. Front. Cell. Infect. Microbiol..

[7] Wang F.Z., Cui Q.R., Zeng Y.N., Chen P. (2020). Intestinal flora-an important participant in liver diseases. J. South Med. Univ..

[8] Zhao M.Q., Wang J., Cui N.L., Zhang X.Q. (2020). Research progress on the relationship between SCFAs and intestinal diseases. Chin. J. Modern Med..

[9] Zhou D., Fan J.G. (2016). Study on the effect of intestinal flora-SCFAs in metabolic diseases. Chin. J. Gastroenterol. Hepatol..

[10] Wu J.X. (2019). Study on the Efficacy and Mechanism of Mussel Polysaccharide on NAFLD Based on “Intestinal Flora-Intestinal-Liver Axis”.

[11] Jin P.F. (2015). Effect and Mechanism of Probiotics on LPS Induced Liver Injury and Expression of Inflammatory Factors in Mice.

[12] Yu J.M., Xie Q.X., Xu H., Qi X.Y., Chen Z., Xu H.Y., Gu W. (2016). Effects of compound microecological preparations of Chinese herbal medicine on intestinal flora and liver function in Broilers. China Feed.

[13] Zhang J. (2019). Protective Effect of Okra on Liver and Moderating Effect of Intestinal Flora.

[14] Zhu Q., Wang X.G., Wang Q., Yuan D.S. (2017). Effect of Xiaozhi Decoction on main intestinal flora in mice with nonalcoholic steatohepatitis. Chin. J. Exp. Tradit. Med. Form..

[15] He J. (2019). Intervention Effect of Polysaccharides from Different Processed Products of Angelica sinensis on Chicken Liver Injury Induced by Ceftiofur Sodium Combined with LPS.

[16] Qiu J.L., Zhang G.F., Chai Y.N., Han X.Y., Zheng H.T., Li X.F., Duan F., Chen L.Y. (2022). Ligustrazine Attenuates Liver Fibrosis by Targeting miR-145 Mediated Transforming Growth Factor-β/Smad Signaling in an Animal Model of Biliary Atresia. J. Pharmacol. Exp. Ther..

[17] Feng S., Tong H., Gao J.H., Tang S.H., Yang W.J., Wang G.M., Zhou H.Y., Wen S.L. (2021). Anti-inflammation treatment for protection of hepatocytes and amelioration of hepatic fibrosis in rats. Exp. Ther. Med..

[18] Xu Q., Xu J., Zhang K., Zhong M., Cao H., Wei R., Jin L., Gao Y. (2021). Study on the protective effect and mechanism of *Dicliptera chinensis* (L.) Juss (Acanthaceae) polysaccharide on immune liver injury induced by LPS. Biomed. Pharmacother..

[19] Li F., Huang D., Nie S., Xie M. (2019). Polysaccharide from the Seeds of *Plantago asiatica* L. Protect Against Lipopolysaccharide-Induced Liver Injury. J. Med. Food..

[20] Wu L., Zhou K., Yang Z., Li J., Chen G., Wu Q., Lv X., Hu W., Rao P., Ai L. (2022). Monascuspiloin from Monascus-Fermented Red Mold Rice Alleviates Alcoholic Liver Injury and Modulates Intestinal Microbiota. Foods.

[21] Shi J.C., Li J.K., Jia L., Lv J.C., Shang Z.H., Zhang T. (2022). Effects of Fermented Traditional Chinese Medicine on the Growth Performance, Antibody Level and Intestinal Flora of Laying Hens. Chin. Anim. Husban Vet. Med..

[22] Zhang R.J., Pan S.Y., Bai Y.Y., Wang G.Q., Wang S.Y. (2005). Regulation mechanism of microbial feed additive YiShengKang on nutritional metabolism and immune function of Broilers. J. China Agric. Univ..

[23] Zhang Z., Liu C.H., Zhang J.H., Lu Y., Zhang Y., Ma W., Wang C.Q. (2021). Effects of dietary add to N-carbamoyl glutamic acid on intestinal morphological development and cecal microflora of roosters. China Feed.

[24] Liu B. (2017). Analysis of Nutrient Metabolic Rate, Immune Function and Intestinal Flora of Broilers with Different Production Performance.

[25] Xing G.R. (2019). Effects of Bacillus coagulans on Performance, Immunity and Intestinal Health of Late Laying Period.

[26] Li W.Y. (2018). Study on Characteristic Changes of Intestinal Flora in Patients with Nonalcoholic Steatohepatitis Based on TCM Syndrome.

[27] Yang B.W., Sun T.N., Yang C.Y., Xiang H.B., Xiong J., Bao S.T. (2022). Cholestatic liver disease induced by common bile duct ligation promotes the change of intestinal flora. Chin. J. Exp. Surg..

[28] Demirci M., Tokman. H.B., Taner Z., Keskin F.E., Çağatay P., Ozturk B.Y., Özyazar M., Kiraz N., Kocazeybek B.S. (2020). Bacteroidetes and Firmicutes levels in gut microbiota and effects of hosts TLR2/TLR4 gene expression levels in adult type 1 diabetes patients in Istanbul, Turkey. J. Diabetes Complicat..

[29] Cui C., Shen C.J., Jia G., Wang K.N. (2013). Effect of dietary Bacillus subtilis on proportion of Bacteroidetes and Firmicutes in swine intestine and lipid metabolism. Genet. Mol. Res..

[30] Kappler K., Yi L., Smith D.F., Opitz L., Hennet T. (2020). Increased Antibody Response to Fucosylated Oligosaccharides and Fucose-Carrying Bacteroides Species in Crohn’s Disease. Front. Microbiol..

[31] Li P. (2019). Geographical Differences of Intestinal Flora and Their Effects on Disease Models.

[32] Li Y.J. (2016). Effects of Bacillus amyloliquefaciens TL on Growth and Cecal flora in Broilers.

[33] Li H.Y., Wang J., Sheng C.L., Zhao X.N. (2015). Analysis of chronic liver injury and Th1/Th2 cell function changes induced by Helicobacter hepaticus in different strains of mice. Chin. J. Lab. Diagn..

[34] Burich A., Hershberg R., Waggie K., Zeng W.P., Brabb T., Westrich G., Viney J.L., Maggio-Price L. (2001). Helieobaeter-induced inflammatory bowel disease in IL-10 and T Cell-deficient mice. Am. J. Physiol. Gastrointest. Liver Physiol..

[35] Jin Y.Y., Xu B., Wang L.Y., Sun Q.Y., Xi Y.Y., Yuan Y.Z., Wang G.L., Fu C., Li S.Y. (2021). Effects of enzymatic hydrolysis of Artemisia annua combined with Bacillus licheniformis on growth performance and cecal flora of Broilers. J. Anim. Nutr..

[36] Wang Y.F., Lin P., Lu J.M., Zhang M., Li L., Yang X.X., Yu J. (2017). Effects of Polygonum multiflorum and its main component stilbene glycoside on intestinal short chain fatty acid production in nonalcoholic fatty liver rats. Modern Chin. Med..

[37] Judith A.W., Chloé V., Julia W., Phuong L., Adriaan G., Holleboom J.V., Max N., Karine C. (2020). Gut microbiota and human NAFLD: Disentangling microbial signatures from metabolic disorders. Nat. Rev. Gastroenterol. Hepatol..

[38] Wang L.X., Liu Y.H., Zhu J.K., Zhong Y., Li L.S., Xu J.D. (2017). Research progress of short chain fatty acids in disease treatment. World Chin. J. Dig..

[39] Li H., Shi J., Zhao L., Guan J.Q., Liu F., Huo G.C., Li B.L. (2021). Lactobacillus plantarum KLDS1.0344 and Lactobacillus acidophilus KLDS1.0901 Mixture Prevents Chronic Alcoholic Liver Injury in Mice by Protecting the Intestinal Barrier and Regulating Gut Microbiota and Liver-Related Pathways. J. Agric. Food Chem..

[40] Yang L., Zhang B., Guan S.X., Hou L.L., Cheng J., Jiang J.H. (2017). Changes of serum short chain fatty acid levels in NAFLD rats. Acta Univ. Med. Anhui.

[41] Li M., Liu S.X., Wang M.Y., Liu M., Hu H.W., Ding Z.B., Huang Y.K. (2019). Levels of short-chain fatty acids in enterobacteria-related metabolites in the feces of infants with cholestatic hepatopathy. Chin. J. Contemp. Pediatr..

[42] Li H., Wang X.H., Ma Z.Q., Ma W.X., Yang L.P. (2022). Clinical significance of the determination of fecal short-chain fatty acids in patients with nonalcoholic fatty liver disease. J. Clin. Hepatol..

[43] Li Y.T. (2018). Effects of Phytosterol Esters on Intestinal Microflora in NAFLD Rats.

[44] Wang L., Zou L., Li J., Yang H., Yin Y. (2021). Effect of dietary folate level on organ weight, digesta pH, short-chain fatty acid concentration, and intestinal microbiota of weaned piglets. J. Anim. Sci..

[45] Peng Y., Chai M.M., Cui X.P., Wang M., Li Y.Y., Wang Y.H. (2020). Effects of butyric acid additives and their combination with synbiotics on growth performance and intestinal health of Broilers. J. Anim. Nutr..

